# Prognostic significance and postoperative chemoradiotherapy guiding value of mean platelet volume for locally advanced esophageal squamous cell carcinoma patients

**DOI:** 10.3389/fonc.2023.1094040

**Published:** 2023-04-26

**Authors:** Wei Zhang, Hongyuan Jia, Xue Chen, Wei Diao, Xuefeng Leng, Bangrong Cao, Yi Wang, Zhuzhong Cheng, Qifeng Wang

**Affiliations:** ^1^ Department of Nuclear Medicine, Sichuan Clinical Research Center for Cancer, Sichuan Cancer Hospital & Institute, Sichuan Cancer Center, Affiliated Cancer Hospital of University of Electronic Science and Technology of China, Chengdu, China; ^2^ Department of Radiation Oncology, Radiation Oncology Key Laboratory of Sichuan Province, Sichuan Clinical Research Center for Cancer, Sichuan Cancer Hospital & Institute, Sichuan Cancer Center, Affiliated Cancer Hospital of University of Electronic Science and Technology of China, Chengdu, China; ^3^ Department of Thoracic Surgery, Sichuan Clinical Research Center for Cancer, Sichuan Cancer Hospital & Institute, Sichuan Cancer Center, Affiliated Cancer Hospital of University of Electronic Science and Technology of China, Chengdu, China; ^4^ Radiation Oncology Key Laboratory of Sichuan Province, Sichuan Clinical Research Center for Cancer, Sichuan Cancer Hospital & Institute, Sichuan Cancer Center, Affiliated Cancer Hospital of University of Electronic Science and Technology of China, Chengdu, China

**Keywords:** esophageal cancer, prognosis, mean platelet volume, postoperative chemoradiation, guiding

## Abstract

**Objective:**

To investigate the predicting prognosis and guiding postoperative chemoradiotherapy (POCRT) value of preoperative mean platelet volume (MPV) in patients with locally advanced esophageal squamous cell carcinoma (LA-ESCC).

**Methods:**

We proposed a blood biomarker, MPV, for predicting disease-free survival (DFS) and overall survival (OS) in LA-ESCC patients who underwent surgery (S) alone or S+POCRT. The median cut-off value of MPV was 11.4 fl. We further evaluated whether MPV could guide POCRT in the study and external validation groups. We used multivariable Cox proportional hazard regression analysis, Kaplan–Meier curves, and log-rank tests to ensure the robustness of our findings.

**Results:**

In the developed group, a total of 879 patients were included. MVP was associated with OS and DFS defined by clinicopathological variables and remained an independent prognostic factor in the multivariate analysis (*P* = 0.001 and *P* = 0.002, respectively). For patients with high MVP, 5-year OS and 0DFS were significantly improved compared to those with low MPV (*P* = 0.0011 and *P* = 0.0018, respectively). Subgroup analysis revealed that POCRT was associated with improved 5-year OS and DFS compared with S alone in the low-MVP group (*P* < 0.0001 and *P* = 0.0002, respectively). External validation group analysis (n = 118) showed that POCRT significantly increased 5-year OS and DFS (*P* = 0.0035 and *P* = 0.0062, respectively) in patients with low MPV. For patients with high MPV, POCRT group showed similar survival rates compared with S alone in the developed and validation groups.

**Conclusions:**

MPV as a novel biomarker may serve as an independent prognosis factor and contribute to identifying patients most likely to benefit from POCRT for LA-ESCC.

## 1 Introduction

Esophageal cancer is a malignant disease with high morbidity and mortality rates worldwide (1), and half of all attributable deaths and incidences occur in China (2). Clinical treatment mainly depends on the clinical stage and pathological type to guide comprehensive treatment (3). For patients with locally advanced esophageal squamous cell carcinoma (LA-ESCC) (T3-4N0 and T1-4N1-3M0), postoperative adjuvant radiotherapy and chemotherapy are recommended (4–7). However, there are still significant differences in treatment outcomes even when patients receive the same combination of treatment patterns under the same staging conditions (8, 9). These results indicate that the current clinicopathological risk stratification model is not an appropriate guide for precise treatment. Some patients do not benefit from postoperative adjuvant chemoradiotherapy, but experience shortened survival due to side effects. There is a critical need for additional prognostic and/or predictive biomarkers beyond the current staging system, which may be used to better inform prognosis and guide treatment strategies (10, 11).

Over the past few decades, increased interest has been found in establishing novel non-invasive predictive biomarkers from hematological serological parameters for various tumors, such as: carcinoembryonic antigen, C-reactive protein, and platelet-related parameters (12, 13). With the ease obtained from routine blood tests, the platelet-related parameters attracted numbers of considerable attention as prognostic indicators in various cancer, including esophageal cancer. Increase numbers of evidence have revealed that platelet activation plays a vital role in tumor growth, invasion, and metastasis (14, 15). As an indicator of platelet activation, mean platelet volume (MPV), was regarded as closely associated with thromboembolism in patients with ischemic stroke, myocardial infarction, and cerebrovascular thromboembolism (16). Recent research has found that MPV levels were significantly higher in colorectal cancer, ovarian cancer, gastric cancer, hepatocellular carcinoma and papillary thyroid carcinomas than in healthy subjects, indicating increased MPV level may serve as an independent prognostic factor for cancer patients (17).

In esophagus cancer, however, the prognosis value of MPV has not yet been comprehensively investigated. He et al. found that decreased MPV is significantly associated with poor prognosis in esophageal squamous cell carcinoma patients (18). However, the results of a recent meta-analysis included 3 studies indicating that MPV were non-independent prognostic factors for OS in patients with esophageal squamous cell carcinoma (19). Therefore, this study aimed to evaluate the relationship between preoperative MPV and long-term survival in patients with LA-ESCC. Moreover, another further aim was to investigate the guiding treatment value of MPV to identify LA-ESCC patients who are most likely to benefit from postoperative adjuvant chemoradiotherapy by developed and external validation group.

## 2 Materials and methods

### 2.1 Patient selection

**Figure 1:**
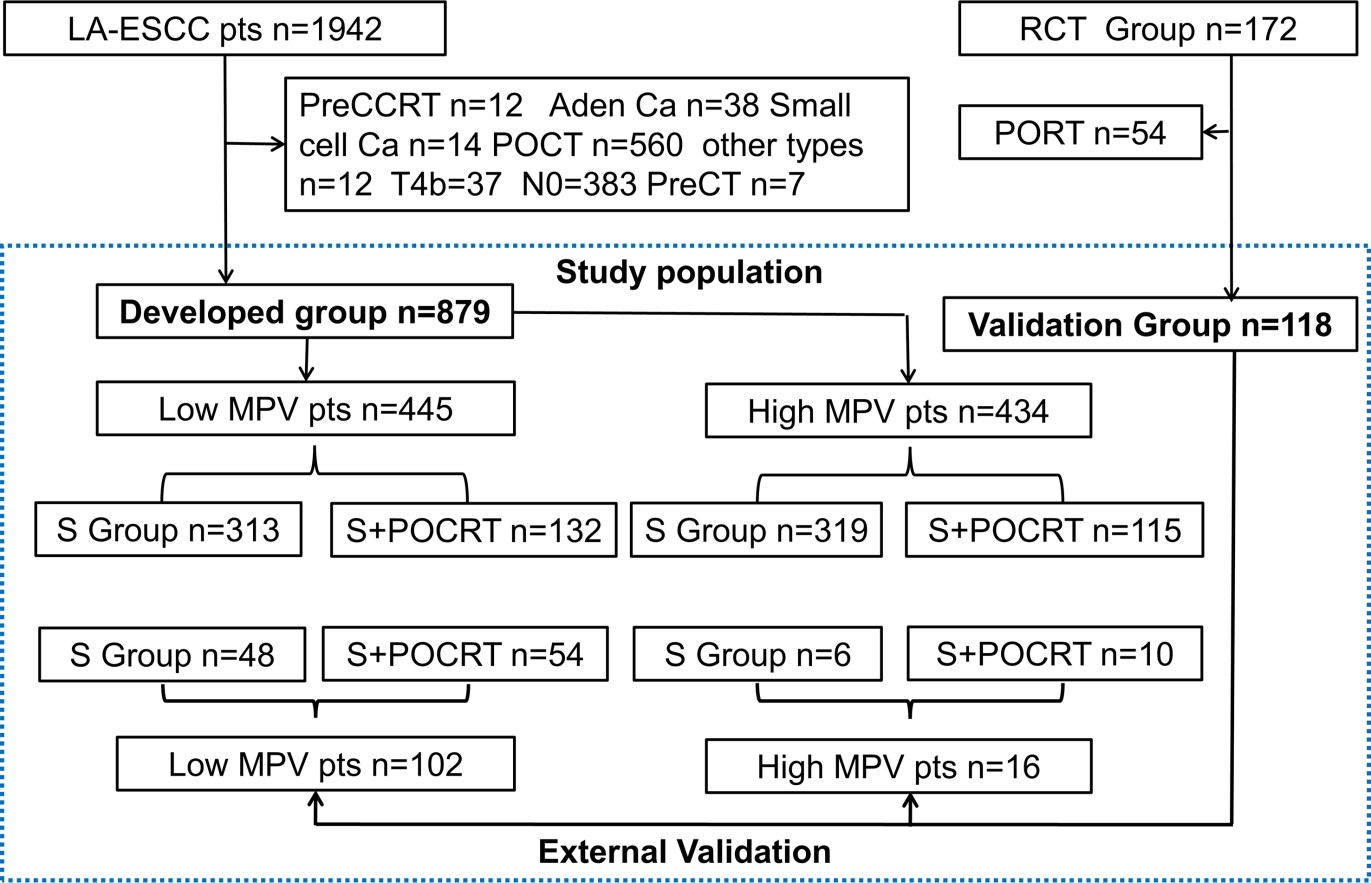
The study flow diagram. LA-ESCC, local advanced esophageal squamous cell carcinoma; preCCRT, preoperative concurrent chemoradiation; Ca, carcinoma; MPV, mean platelet volume; POCRT, postoperative chemoradiotherapy; PORT, postoperative radiotherapy; SA, surgery alone; TNM, Tumor Nodes Metastasis; VATS, Video-assisted thoracoscopic surgery. RCT, randomized clinical trial.

Between January 2008 and December 2017, we retrospectively identified 1,942 patients with LA-ESCC who underwent esophagectomy with curative intent, which served as developed group. The eligibility criteria were: 1. histologically proven thoracic LA-ESCC; 2. curative R0 resection; 3. the surgical technique used was standard McKeown esophagectomy or Ivor-Lewis esophagectomy; 4. pathologically proven stage IIB–IVa ESCC based on the 8th edition American Joint Committee on Cancer (AJCC) staging system;5. age ≥18 years with a Karnofsky performance status (KPS) ≥70; 6. adequate bone marrow, renal, and hepatic functions; 7. routine blood test one week before the operation, including MPV; and 8. fit for postoperative chemoradiation. The exclusion criteria were cervical esophageal tumors, preoperative chemoradiation or chemotherapy, postoperative chemotherapy, or radiotherapy alone, and other pathological types, such as adenocarcinoma and small cell carcinoma. The study protocol was approved by the appropriate institutional ethics review board (SCCHEC-02-2020-015) and that of Cancer Hospital, Chinese Academy of Medical Sciences (ID:14-090/880). For external validation, we selected 172 patients with lymph node-positive or stage III ESCC who were enrolled in a prospective phase III randomized controlled trial (20) from October 2014 through December 2019. A flow chart of patient selection is shown in **Figure 1**.

### 2.2 Surgery

All patients received intravenous and inhalation-based general anesthesia. The surgical techniques used were standard McKeown esophagectomy (n=702) and Ivor-Lewis esophagectomy (n=177). Surgical approaches included minimally invasive esophagectomy (n=439) and open esophagectomy (n=440). Pathological staging of the surgical specimen was based on the 8th edition of the TNM classification for esophageal cancer (21). The validation treatment has been previously described in detail by Ni et al. (20).

### 2.3 Postoperative chemoradiation

Radiotherapy began 4–10 weeks after surgery. Computed tomography was used to identify anatomical landmarks and delineate mediastinal lymph node stations. The clinical target volume (CTV) was defined as the tumor bed and the high-risk lymphatic drainage area. Anastomosis was included in the CTV for patients with upper thoracic tumors and those with an insufficient proximal margin (<3 cm). Radiotherapy involved a total dose of 50–54 Gy delivered to 95% of the planning target volume in 25–30 fractions (five fractions/week for 5–6 weeks).

Platinum drugs were mainly used for chemotherapy during postoperative chemoradiotherapy (POCRT). Cisplatin-based, nedaplatin-based, oxaliplatin-based, and carboplatin-based regimens were used for 92, 30, 30, and 54 patients, respectively. In addition, Tegafur, Gimeracil and Oteracil Porassium Capsules treatment was administered to 41 patients.

### 2.4 Statistic methods

The correlation between preoperative MPV values and the clinical pathology of patients with LA-ESCC was analyzed. Patients were divided into four groups according to the median MPV value (low ≤ 11.4 fl v.s. high >11.4 fl) and different treatments (surgery alone (S alone) or S+POCRT). The clinicopathological indicators of different MPV groups (low and high) were compared and analyzed using the Chi-square test or Fisher’s exact test. The univariate analysis included sex, age, KPS score, tumor length, tumor location, tumor differentiation, lymphovascular invasion, nerve invasion, number of lymph node dissections, pathological TNM stage, and POCRT. Multivariate Cox regression analysis was performed for variables with *P* < 0.1 in the univariate analysis. Overall survival (OS) and disease-free survival (DFS) of patients with low- and high-MVP were compared using the Kaplan–Meier method, and the log-rank test was used for patients that underwent S alone. Kaplan–Meier curves and log-rank tests were used for stratification according to the MPV group to compare the survival difference between the S+POCRT and S alone group. For external validation, we selected patients from a prospective randomized stage III study to evaluate whether POCRT improved OS and DFS compared with S alone in the low MPV group. Statistical analysis was performed using R software V. 3.5.1 (https://www.Rproject.org/). Statistical significance was set at *P* < 0.05.

## 3 Results

### 3.1 Patient characteristics

In this study, a total of 1,942 patients were identified as LA-ESCC for developed group, and finally, a total of 879 patients (632 for S alone and 247 for S+POCRT) were included for further analysis. After excluding the S plus postoperative radiotherapy group (n = 54), finally, a total of 118 patients (S alone (n = 54) and S+POCRT (n= 64)) were included for further validation analysis (**Table S1**). Based on the median MPV value of 11.4 fl, the patients were divided into two groups (low MPV, n = 445; high MPV group, n = 434). **Figure 1** presented the detailed study flow. **Table 1** presented the detailed clinicopathological characteristics, in which 62.9% (553) of patients were men with a median age of 64 (range, 39–85) years and a median follow-up period of 42.5 (range, 24–116) months. The 5-year OS and DFS rates in the overall study cohort were 35.2% and 28.0%, respectively.

**Table 1:** Clinical and pathological factors in different mean platelet volume.

Variables n (%)	Total (n = 879)	Low MPV (n = 445)	High MPV (n = 434)	*P* value
Age(year)				0.292
≤ 65	553 (62.9)	288 (64.7)	265 (61.1)	
>65	326 (37.1)	157 (35.3)	169 (38.9)	
Gender				< 0.001
Male	732 (83.3)	390 (87.6)	342 (78.8)	
Female	147 (16.7)	55 (12.4)	92 (21.2)	
KPS				0.465
90 -100	580 (66.0)	288 (64.7)	292 (67.3)	
70 - 80	299 (34.0)	157 (35.3)	142 (32.7)	
Number of LN resection				0.155
≤1 5	220 (25.0)	121 (27.2)	99 (22.8)	
>15	659 (75.0)	324 (72.8)	335 (77.2)	
Differentiation				0.774
High	142 (16.2)	70 (15.7)	72 (16.6)	
Middle	368 (41.9)	183 (41.1)	185 (42.6)	
Poor	369 (42.0)	192 (43.1)	177 (40.8)	
Location				0.568
Upper	272 (30.9)	139 (31.2)	133 (30.6)	
Middle	436 (49.6)	214 (48.1)	222 (51.2)	
Lower	171 (19.5)	92 (20.7)	79 (18.2)	
Lymphovascular invasion				0.027
Yes	199 (22.6)	115 (25.8)	84 (19.4)	
No	680 (77.4)	330 (74.2)	350 (80.6)	
Neural invasion				0.901
Yes	197 (22.4)	101 (22.7)	96 (22.1)	
No	682 (77.6)	344 (77.3)	338 (77.9)	
Path T stage				0.325
T1	42 (4.8)	22 (4.9)	20 (4.6)	
T2	149 (17.0)	72 (16.2)	77 (17.7)	
T3	566 (64.4)	280 (62.9)	286 (65.9)	
T4a	122 (13.9)	71 (16)	51 (11.8)	
Path N stage				0.039
N0	39 (4.4)	17 (3.8)	22 (5.1)	
N1	460 (52.3)	219 (49.2)	241 (55.5)	
N2	265 (30.1)	138 (31)	127 (29.3)	
N3	115 (13.1)	71 (16)	44 (10.1)	
Treatment				0.333
SA	632 (71.9)	313 (70.3)	319 (73.5)	
SA+POCRT	247 (28.1)	132 (29.7)	115 (26.5)	

MPV, mean platelet volume; KPS, Karnofsky performance score; LN, lymph node; Path, pathology; TNM, Tumor Nodes Metastasis; SA, surgery alone; POCRT, postoperative chemoradiotherapy.

### 3.2 Relationships between MPV, and clinicopathological features

Overall, significant associations were found between the MPV and factors such as sex (*P* < 0.001), lymphovascular invasion (*P* = 0.027), and lymph node metastasis (*P* = 0.039), and no significant differences in age (*P* = 0.292), tumor location (*P* = 0.568), T stage (*P* = 0.325) and treatment (*P* = 0.333) (**Table 1**).

### 3.3 Association between mean platelet volume, treatment regimen, and prognosis

**Figure 2:**
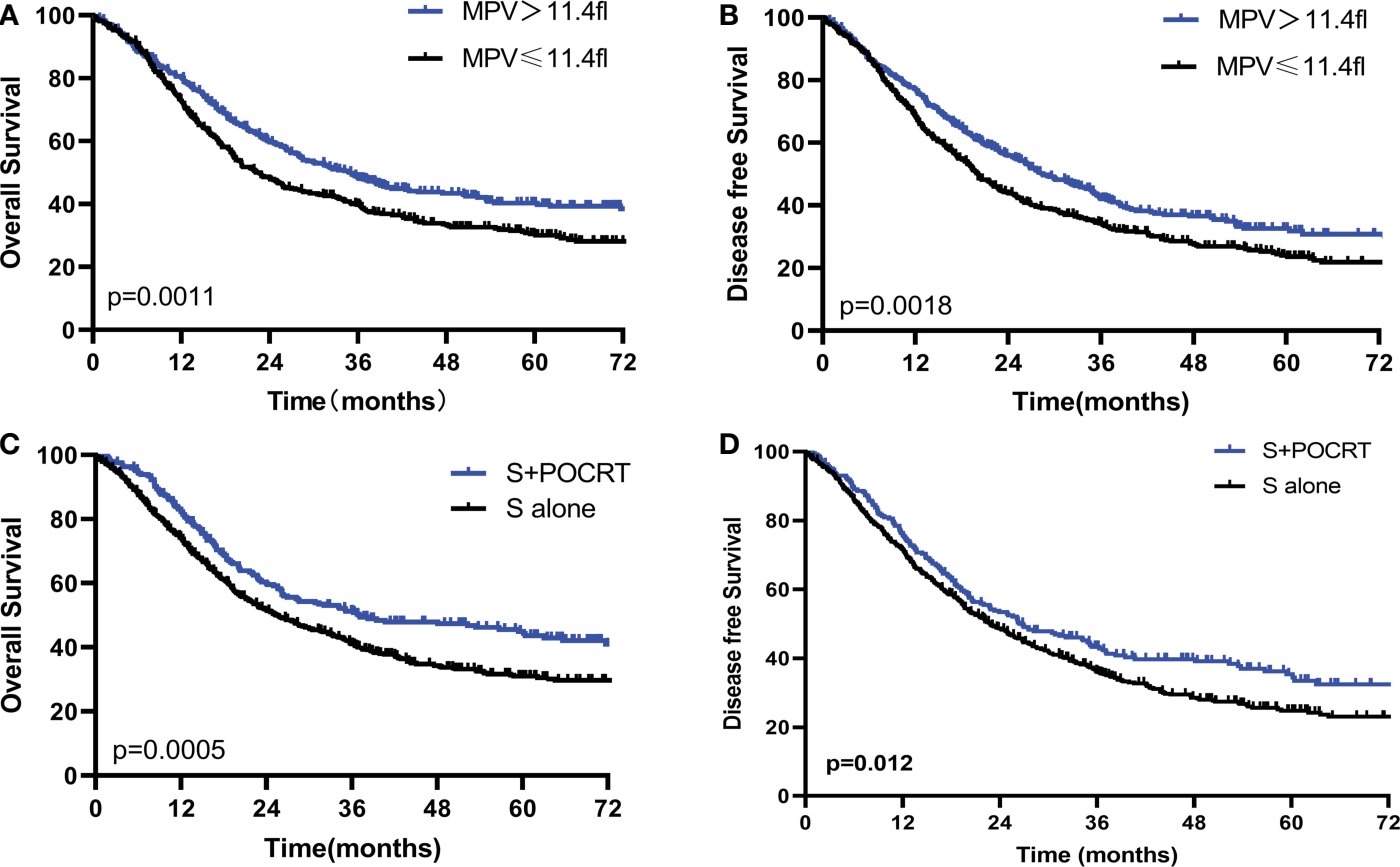
Comparison of overall survival and disease-free survival between high and low MPV **(A, B)**, and POCRT and SA group **(C, D)** in the developed population. POCRT, postoperative chemoradiotherapy; SA, surgery alone; MPV, mean platelet volume.

The Kaplan–Meier curves exhibited that patients with low MPV had a worse OS (P < 0.001, **Figure 2A**) compared with the high MPV group. For patients with high MPV (n = 434), 5-year OS was significantly improved compared to those with low MPV (n = 445) (40.3% vs. 30.4%, *P* = 0.001, **Figure 2A**). Cox multivariate analysis showed that low MPV was associated with worse OS (HR 0.75, 95%CI: 0.64 – 0.89, *P* = 0.001, **Table 2**). Compared with the S alone group, the S+POCRT group had significantly better 5-year OS outcomes (44.9% vs. 31.0%, *P* = 0.0005, **Figure 2C**). Cox multivariate analysis showed that S+POCRT was associated with improved OS (HR 0.71, 95%CI: 0.58 – 0.86, *P* = 0.001, **Table 2**) as independent prognostic factors.

**Table 2:** Multivariable analysis associations between Mean platelet volume and Overall survival and disease-free survival.

Variable	HR for DFS (95% CI)	*P* value	HR for OS (95% CI)	*P* value
Gender				
Male	1		1	
Female	0.7 (0.55~0.88)	0.002	0.69 (0.54~0.88)	0.003
Lymphovascular invasion				
Yes	1		1	
No	0.69 (0.57~0.83)	<0.001	0.69 (0.57~0.84)	<0.001
Number of LN resected				
≤15	1		1	
>15	0.86 (0.72~1.03)	0.111	0.85 (0.7~1.03)	0.092
Path T stage				
T1	1		1	
T2	1.67 (1~2.78)	0.048	2.13 (1.18~3.83)	0.012
T3	2.43 (1.52~3.9)	<0.001	2.98 (1.71~5.18)	<0.001
T4a	2.58 (1.55~4.29)	<0.001	3.4 (1.89~6.11)	<0.001
Path N stage				
N0	1		1	
N1	1.09 (0.69~1.71)	0.721	1.07 (0.66~1.72)	0.795
N2	1.84 (1.16~2.91)	0.009	1.92 (1.19~3.12)	0.008
N3	2.37 (1.46~3.83)	<0.001	2.31 (1.39~3.84)	0.001
MPV group				
low	1		1	
high	0.77 (0.66~0.91)	0.002	0.75 (0.64~0.89)	0.001
Treatment				
SA	1		1	
SA+POCRT	0.79 (0.66~0.95)	0.012	0.71 (0.58~0.86)	0.001

DFS, disease-free survival; OS, overall survival; HR, hazard ratio; CI, confidence interval; LN, lymph node; Path, pathology; TNM, Tumor Nodes Metastasis; SA, surgery alone; POCRT, postoperative chemoradiotherapy.

For DFS, patients with low MPV had a worse OS compared with the high MPV group (5-year DFS: 32.6% vs. 24.1%, *P* =0.001, **Figure 2B**). Cox multivariate analysis showed that low MVP was associated with worse OS (HR 0.77, 95%CI: 0.66 – 0.91, *P* = 0.002, **Table 2**). Compared with the S alone group, the S+POCRT group had significantly better 5-year DFS outcomes (35.3% vs. 24.7%, *P* = 0.012, **Figure 2D**). Cox multivariate analysis showed that S+POCRT was associated with improved DFS (HR 0.79, 95%CI: 0.66 – 0.95, *P* = 0.001, **Table 2**) as independent prognostic factors.

### 3.4 Association between MPV signature and the benefit of POCRT

**Figure 3:**
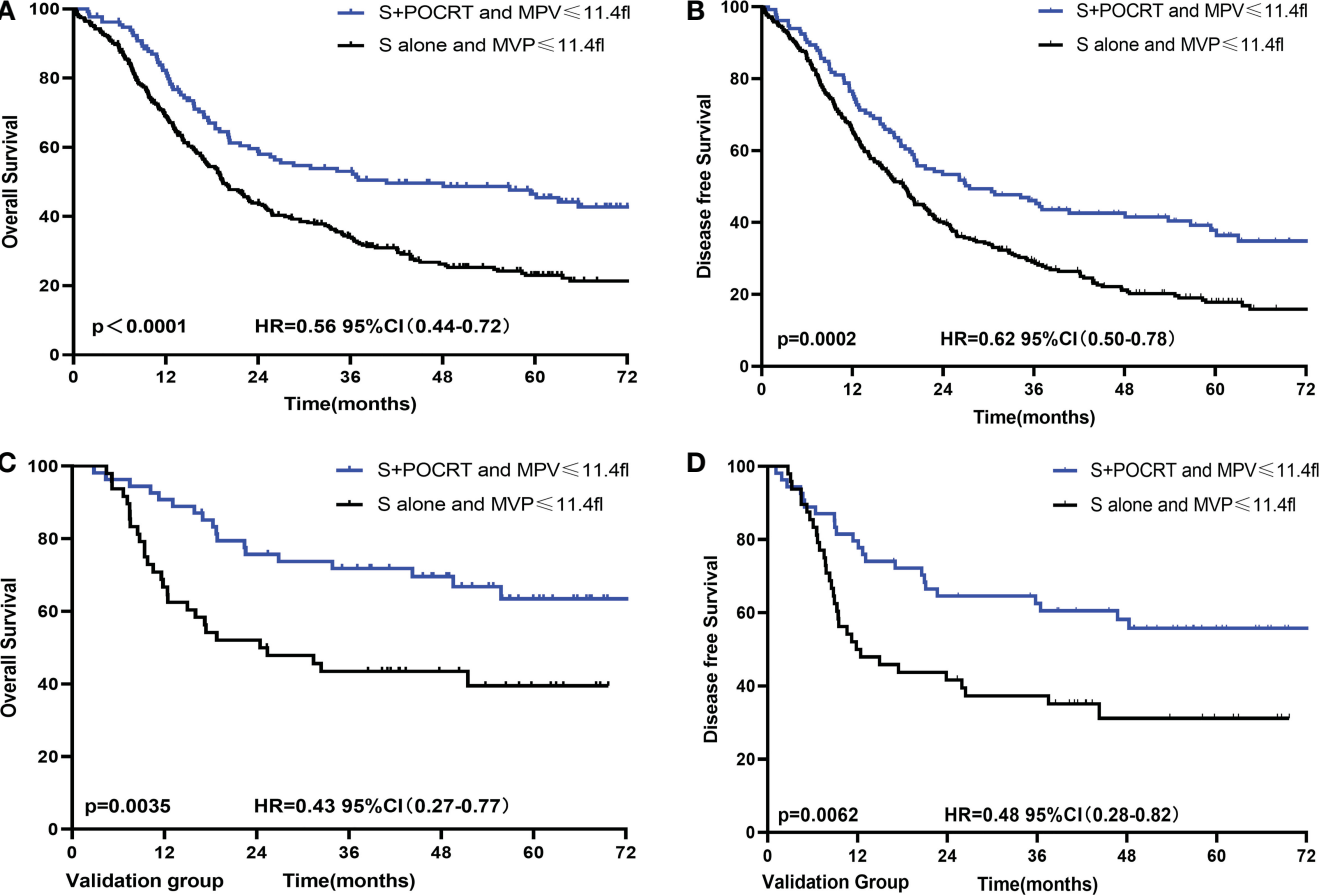
Comparison of overall survival and disease-free survival between POCRT and SA group for low MPV in developed **(A, B)** and validation **(C, D)** population. CI, confidence interval; HR, hazard ratio; POCRT, postoperative chemoradiotherapy; SA, surgery alone; MPV, mean platelet volume.

To further evaluate the value of MPV guidance in POCRT for LA-ESCC, patients were divided into four groups according to treatment methods (S and S+POCRT) and MPV (**Table S2**). For patients with low MPV, the S+POCRT group had significantly better 5-year OS and DFS outcomes (OS: 46.5%; *P* < 0.0001, **Figure 3A**; DFS: 37.8%; *P* = 0.0002, **Figure 3B**) than that of the S alone group (OS: 23.0%; DFS: 17.8%). Cox multivariate analysis showed that S+POCRT was associated with improved OS and DFS as independent prognostic factors, independent of pTNM stage (both *P* < 0.001, **Table S3**). For patients with high MPV, S+POCRT had similar 5-year OS and DFS outcomes (OS 39.2% vs. 43.0%, *P* = 0.481; DFS 32.2% vs. 36.6%, *P* = 0.386, **Figures S1A–D**), relative to the S alone group. Cox multivariate analysis showed that S+POCRT was not associated with OS and DFS (**Table S4**; **Figures S1A, B**) in patients with high MPV.

### 3.5 External validation of the benefit of POCRT among patients from a prospective clinical trial

The external validation group comprised patients from a prospective clinical trial. These patients were divided into different MPV groups (cutoff value = 11.4 fl, **Table 3**). For patients with a low MPV, the S+POCRT group had significantly better OS outcomes (5-year OS: 63.4% vs. 39.5%; *P* = 0.0035; **Figure 3C**) than did patients with a high MPV. Compared with the S alone group (5-year DFS: 31.2%), the S+POCRT group had better DFS outcomes (5-year DFS: 55.8%; *P* = 0.0062; **Figure 3D**). In the subgroup analysis of patients with low MPV, Cox multivariate analysis showed that S+POCRT was associated with OS and DFS as independent prognostic factors (*P* = 0.014; **Table S5**). For patients with high MPV, relative to the S alone group, S+POCRT had similar 5-year OS and DFS outcomes (**Figures S1C, D**).

**Table 3:** Clinicopathological factors in developed and validation group with low mean platelet volume.

Variables, n (%)	Total (n = 445)	Low MPV		P value	Validation (n = 102)	Low MPV		P value
		SA (n = 313)	POCRT (n = 132)			SA (n = 48)	POCRT (n = 54)	
Age(year)				< 0.001				0.018
≤ 65	288 (64.7)	181 (57.8)	107 (81.1)		90 (88.2)	38 (79.2)	52 (96.3)	
>65	157 (35.3)	132 (42.2)	25 (18.9)		12 (11.8)	10 (20.8)	2 (3.7)	
Gender				0.953				1
Male	390 (87.6)	275 (87.9)	115 (87.1)		93 (91.2)	44 (91.7)	49 (90.7)	
Female	55 (12.4)	38 (12.1)	17 (12.9)		9 (8.8)	4 (8.3)	5 (9.3)	
KPS				0.84				0.188
90-100	288 (64.7)	204 (65.2)	84 (63.6)		42 (41.2)	16 (33.3)	26 (48.1)	
70-80	157 (35.3)	109 (34.8)	48 (36.4)		60 (58.8)	32 (66.7)	28 (51.9)	
Number of LN resection				0.543				0.249
≤15	121 (27.2)	82 (26.2)	39 (29.5)		7 (6.9)	5 (10.4)	2 (3.7)	
>15	324 (72.8)	231 (73.8)	93 (70.5)		95 (93.1)	43 (89.6)	52 (96.3)	
Differentiation				0.881				0.371
High	70 (15.7)	51 (16.3)	19 (14.4)		6 (5.9)	4 (8.3)	2 (3.7)	
Middle	183 (41.1)	128 (40.9)	55 (41.7)		60 (58.8)	25 (52.1)	35 (64.8)	
Poor	192 (43.1)	134 (42.8)	58 (43.9)		36 (35.3)	19 (39.6)	17 (31.5)	
Location				0.179				0.368
Upper	139 (31.2)	101 (32.3)	38 (28.8)		6 (5.9)	3 (6.2)	3 (5.6)	
Middle	214 (48.1)	142 (45.4)	72 (54.5)		33 (32.4)	12 (25)	21 (38.9)	
Lower	92 (20.7)	70 (22.4)	22 (16.7)		63 (61.8)	33 (68.8)	30 (55.6)	
Lymphovascular invasion				0.702				0.597
Yes	115 (25.8)	83 (26.5)	32 (24.2)		45 (44.1)	23 (47.9)	22 (40.7)	
No	330 (74.2)	230 (73.5)	100 (75.8)		57 (55.9)	25 (52.1)	32 (59.3)	
Neural invasion				0.542				< 0.001
Yes	101 (22.7)	74 (23.6)	27 (20.5)		23 (22.5)	20 (41.7)	3 (5.6)	
No	344 (77.3)	239 (76.4)	105 (79.5)		79 (77.5)	28 (58.3)	51 (94.4)	
Path T stage				0.294				0.697
T1	22 (4.9)	13 (4.2)	9 (6.8)		14 (13.7)	5 (10.4)	9 (16.7)	
T2	72 (16.2)	55 (17.6)	17 (12.9)		13 (12.7)	5 (10.4)	8 (14.8)	
T3	280 (62.9)	199 (63.6)	81 (61.4)		68 (66.7)	35 (72.9)	33 (61.1)	
T4a	71 (16.0)	46 (14.7)	25 (18.9)		7 (6.9)	3 (6.2)	4 (7.4)	
Path N stage				0.401				0.835
N0	17 (3.8)	10 (3.2)	7 (5.3)		4 (3.9)	1 (2.1)	3 (5.6)	
N1	219 (49.2)	152 (48.6)	67 (50.8)		60 (58.8)	30 (62.5)	30 (55.6)	
N2	138 (31.0)	96 (30.7)	42 (31.8)		31 (30.4)	14 (29.2)	17 (31.5)	
N3	71 (16.0)	55 (17.6)	16 (12.1)		7 (6.9)	3 (6.2)	4 (7.4)	

MPV, mean platelet volume; SA, surgery alone; POCRT, postoperative chemoradiotherapy; KPS, Karnofsky performance score; LN, lymph node; Path, pathology; TNM, Tumor Nodes Metastasis.

## 4 Discussion

Esophagus cancer is a highly lethal malignancy (1). For LA-ESCC, comprehensive treatment is the main method used to improve the therapeutic effect. Clinical stage and pathological type are mainly tools for guiding treatment. Despite receiving similar treatment patterns and having similar staging conditions, patients may still experience significant differences in treatment outcomes. This is largely due to the fact that pathological staging does not consider the tumor microenvironment. Considering the limitation of pathology in guiding treatment, lots of novel non-invasive predictive biomarkers were developed and used to guide treatment strategies. The MPV is an indicator of platelet activation and is considered a novel biomarker for predicting the prognosis of various cancer, which a decreased MPV is significantly associated with a poor prognosis (18). For esophagus cancer, however, the prognosis value of MPV is still unclear. In this study, we found MPV can serve as an independent prognostic factor for LA-ESCC, and patients with low MPV were significantly associated with worse 5-year OS (HR 0.75, 95%CI: 0.64 – 0.89, *P* = 0.001) and DFS (HR 0.77, 95%CI: 0.66 – 0.91, *P* = 0.002).

Decreased MPV was found associated with poor prognosis in LA-ESCC. However, the cause of decreased MPV in LA-ESCC remains unclear (15, 22). The main cause of low MPV, an important indicator of platelet function, may be chronic inflammation which caused the excessive platelet consumption (17). Consistent with our findings, Shen et al. (23) also found that a lower MPV was associated with poor prognosis in esophageal cancer, although they used a different cutoff value, which may be attributable to differences in pathological staging and number of patients. Both our study and Shen’s study found a significant correlation between low MPV and lymphovascular infiltration and lymph node metastasis. Different MPVs have different prognostic values for different types of cancer (17). For gastric cancer, Shen et al. reported that increased MPV was associated with poor prognosis (23), whereas high MPV in blood tumors, renal cancer, hepatocellular carcinoma, and lung cancer is associated with advanced-stage or an unfavorable disease-like thrombotic state (24, 25).

With significantly improved overall survival, postoperative adjuvant radiotherapy and chemotherapy are the recommended treatment strategy for LA-ESCC (20, 26, 27). However, the treatment results under the pathological staging guide are controversial (5, 7, 8). A meta-analysis including 11 articles and a total of 2,047 ESCC patients found that the 3-year OS between adjuvant chemotherapy (n = 887) and S alone (n = 1160) groups) was not significantly different (risk ratio = 0.89, 95%CI, 0.72 -1.09; *P*= 0.25) (9). In this study, we found that compared with the S alone group, the S+POCRT group had significantly improved 5-year OS outcomes (HR 0.71, 95%CI: 0.58 – 0.86, P = 0.001) and DFS (HR 0.79, 95%CI: 0.66 – 0.95, *P* = 0.001). Therefore, standard adjuvant treatment remains controversial. Individualized and accurate identification of patients who can benefit from adjuvant radiotherapy and chemotherapy is important for improving the curative effect.

Another important finding of this study is that the MPV level can be used as a guide for selecting treatment for LA-ESCC patients. Patients with low MPV may experience improved 5-year OS and DFS outcomes with POCRT, while patients with high MPV may have similar 5-year OS (*P* = 0.481) and DFS outcomes (*P* = 0.386) as the S alone group but underwent additional side effects of POCRT. A similar result was found in a limited prospective clinical trial. Therefore, LA-ESCC patients with high MPV which indicates low risk for recurrence and metastasis, avoid choosing the postoperative radiotherapy therapeutic schedule. A small side effects treatment scheme, such as the Nivolumab which has proven curative effects by the checkmate 577 study, may be an optimal treatment option (28). For patients who did not achieve pathological complete response after receiving neoadjuvant chemoradiation, postoperative adjuvant immunotherapy can significantly improve disease-free progression.

This study has some limitations. First, it was a retrospective study. A large sample size and multicentric prospective study is needed to carry out to investigate the prognostic significance and POCRT guiding value of MPV in LA-ESCC. Second, the optimal cut-off value in this study was determine by median MPV value (11.4 fl) in developed group and 11.4 fl was directly used as the cut-off value in the external validation group, which may lead to inaccuracy grouping. A more accurate methods are needed to determine the optimal cut-off value in the further studies. Third, this study cannot represent early esophageal cancer, nor can it guide preoperative radiotherapy and chemotherapy, which are all aspects that must be studied in the future.

## 5 Conclusions

In conclusion, MPV can serve as an independent prognostic factor for LA-ESCC. Patients with low MPV were significantly associated with poor prognoses. Additionally, MPV level can contribute to treatment selection for LA-ESCC patients, which patients with low MPV may benefit from POCRT, resulting in an improved 5-year survival rate. Although MPV is a non-specific marker, this noninvasive, convenient, and inexpensive biomarker may complement the present pTNM staging in guiding treatment.

## Data availability statement

The original contributions presented in the study are included in the article/**Supplementary Material**. Further inquiries can be directed to the corresponding authors.

## Ethics statement

The studies involving human participants were reviewed and approved by The Sichuan Cancer Hospital ethics committee. The patients/participants provided their written informed consent to participate in this study.

## Author contributions

In this paper, WZ and HJ conceived and designed the study. WZ and HJ performed data collection, manuscript preparation. XC performed manuscript preparation. WD performed manuscript improved. XL, BC and YW performed statistical analysis, and data interpretation. ZC and QW guaranteed the integrity of the entire study and revised the manuscript. All authors contributed to the article and approved the submitted version.
