# Supplementary material for: Prognostic significance and postoperative chemoradiotherapy guiding value of mean platelet volume for locally advanced esophageal squamous cell carcinoma patients
Source: Front Oncol. 2023 Apr 26;13:1094040. doi: 10.3389/fonc.2023.1094040 (PMC10171920; doi:10.3389/fonc.2023.1094040)
Supplement: Supplementary file 1 [file DataSheet_1.docx]

| Table S1：clinical and pathological factors for different MPV and treatment in develop group | | | | | | |
| --- | --- | --- | --- | --- | --- | --- |
| Variables | Total  (n = 879) | Low MPV |  | High MPV |  | p |
|  |  | S  (n = 313) | S+POCRT  (n = 132) | S  (n = 319) | POCRT  (n = 115) |  |
| Age(year) |  |  |  |  |  | < 0.001 |
| ≤65 | 553 (62.9) | 181 (57.8) | 107 (81.1) | 182 (57.1) | 83 (72.2) |  |
| ＞65 | 326 (37.1) | 132 (42.2) | 25 (18.9) | 137 (42.9) | 32 (27.8) |  |
| Gender |  |  |  |  |  | < 0.001 |
| Male | 732 (83.3) | 275 (87.9) | 115 (87.1) | 244 (76.5) | 98 (85.2) |  |
| Female | 147 (16.7) | 38 (12.1) | 17 (12.9) | 75 (23.5) | 17 (14.8) |  |
| KPS |  |  |  |  |  | 0.032 |
| 90-100 | 580 (66.0) | 204 (65.2) | 84 (63.6) | 227 (71.2) | 65 (56.5) |  |
| 70-80 | 299 (34.0) | 109 (34.8) | 48 (36.4) | 92 (28.8) | 50 (43.5) |  |
| Number of LN resection |  |  |  |  |  | 0.107 |
| ≤15 | 220 (25.0) | 82 (26.2) | 39 (29.5) | 80 (25.1) | 19 (16.5) |  |
| ＞15 | 659 (75.0) | 231 (73.8) | 93 (70.5) | 239 (74.9) | 96 (83.5) |  |
| Differentiation |  |  |  |  |  | 0.99 |
| High | 142 (16.2) | 51 (16.3) | 19 (14.4) | 54 (16.9) | 18 (15.7) |  |
| Middle | 368 (41.9) | 128 (40.9) | 55 (41.7) | 135 (42.3) | 50 (43.5) |  |
| Poor | 369 (42.0) | 134 (42.8) | 58 (43.9) | 130 (40.8) | 47 (40.9) |  |
| Location |  |  |  |  |  | 0.214 |
| Upper | 272 (30.9) | 101 (32.3) | 38 (28.8) | 93 (29.2) | 40 (34.8) |  |
| Middle | 436 (49.6) | 142 (45.4) | 72 (54.5) | 172 (53.9) | 50 (43.5) |  |
| Lower | 171 (19.5) | 70 (22.4) | 22 (16.7) | 54 (16.9) | 25 (21.7) |  |
| Lymphovascular invasion |  |  |  |  |  | 0.124 |
| 1 | 199 (22.6) | 83 (26.5) | 32 (24.2) | 60 (18.8) | 24 (20.9) |  |
| 2 | 680 (77.4) | 230 (73.5) | 100 (75.8) | 259 (81.2) | 91 (79.1) |  |
| Neural invasion |  |  |  |  |  | 0.319 |
| 1 | 197 (22.4) | 74 (23.6) | 27 (20.5) | 64 (20.1) | 32 (27.8) |  |
| 2 | 682 (77.6) | 239 (76.4) | 105 (79.5) | 255 (79.9) | 83 (72.2) |  |
| PathTstage, n (%) |  |  |  |  |  | 0.498 |
| T1 | 42 ( 4.8) | 13 (4.2) | 9 (6.8) | 16 (5) | 4 (3.5) |  |
| T2 | 149 (17.0) | 55 (17.6) | 17 (12.9) | 59 (18.5) | 18 (15.7) |  |
| T3 | 566 (64.4) | 199 (63.6) | 81 (61.4) | 207 (64.9) | 79 (68.7) |  |
| T4a | 122 (13.9) | 46 (14.7) | 25 (18.9) | 37 (11.6) | 14 (12.2) |  |
| PathNstage, n (%) |  |  |  |  |  | 0.125 |
| N0 | 39 ( 4.4) | 10 (3.2) | 7 (5.3) | 17 (5.3) | 5 (4.3) |  |
| N1 | 460 (52.3) | 152 (48.6) | 67 (50.8) | 183 (57.4) | 58 (50.4) |  |
| N2 | 265 (30.1) | 96 (30.7) | 42 (31.8) | 90 (28.2) | 37 (32.2) |  |
| N3 | 115 (13.1) | 55 (17.6) | 16 (12.1) | 29 (9.1) | 15 (13) |  |

Abbreviations:KPS, Karnofsky performance score; LVI, lymphovascular invasion; POCRT, postoperative chemoradiotherapy; SA, surgery alone; TNM, Tumor Nodes Metastasis; LN：lymph node; Path:pathology ; MPV: mean palalet volume;

Table S2：clinicalpathological factors for different MPV and treatment in validation group

| Variables | Total (n = 118) | Low MPV (n = 102) | High MPV (n = 16) | p |
| --- | --- | --- | --- | --- |
| Age(year) |  |  |  | 0.229 |
| ≤65 | 102 (86.4) | 90 (88.2) | 12 (75) |  |
| ＞65 | 16 (13.6) | 12 (11.8) | 4 (25) |  |
| Gender |  |  |  | 0.208 |
| Male | 106 (89.8) | 93 (91.2) | 13 (81.2) |  |
| Female | 12 (10.2) | 9 (8.8) | 3 (18.8) |  |
| KPS |  |  |  | 0.185 |
| 90-100 | 52 (44.1) | 42 (41.2) | 10 (62.5) |  |
| 70-80 | 66 (55.9) | 60 (58.8) | 6 (37.5) |  |
| Number of LN resection |  |  |  | 0.351 |
| ≤15 | 9 ( 7.6) | 7 (6.9) | 2 (12.5) |  |
| ＞15 | 109 (92.4) | 95 (93.1) | 14 (87.5) |  |
| Differentiation |  |  |  | 0.741 |
| High | 7 ( 5.9) | 6 (5.9) | 1 (6.2) |  |
| Middle | 68 (57.6) | 60 (58.8) | 8 (50) |  |
| Poor | 43 (36.4) | 36 (35.3) | 7 (43.8) |  |
| Location |  |  |  | 0.175 |
| Upper | 6 ( 5.1) | 6 (5.9) | 0 (0) |  |
| Middle | 42 (35.6) | 33 (32.4) | 9 (56.2) |  |
| Lower | 70 (59.3) | 63 (61.8) | 7 (43.8) |  |
| Lymphovascular invasion |  |  |  | 1 |
| No | 66 (55.9) | 57 (55.9) | 9 (56.2) |  |
| Yes | 52 (44.1) | 45 (44.1) | 7 (43.8) |  |
| Neural invasion |  |  |  | 0.517 |
| No | 93 (78.8) | 79 (77.5) | 14 (87.5) |  |
| Yes | 25 (21.2) | 23 (22.5) | 2 (12.5) |  |
| PathTstage, n (%) |  |  |  | 1 |
| T1 | 16 (13.6) | 14 (13.7) | 2 (12.5) |  |
| T2 | 15 (12.7) | 13 (12.7) | 2 (12.5) |  |
| T3 | 79 (66.9) | 68 (66.7) | 11 (68.8) |  |
| T4a | 8 ( 6.8) | 7 (6.9) | 1 (6.2) |  |
| PathNstage, n (%) |  |  |  | 0.413 |
| N0 | 4 ( 3.4) | 4 (3.9) | 0 (0) |  |
| N1 | 69 (58.5) | 60 (58.8) | 9 (56.2) |  |
| N2 | 35 (29.7) | 31 (30.4) | 4 (25) |  |
| N3 | 10 ( 8.5) | 7 (6.9) | 3 (18.8) |  |
| Treatment |  |  |  | 0.657 |
| S alone | 64 (54.2) | 54 (52.9) | 10 (62.5) |  |
| POCRT | 54 (45.8) | 48 (47.1) | 6 (37.5) |  |

Abbreviations:KPS, Karnofsky performance score; LVI, lymphovascular invasion; POCRT, postoperative chemoradiotherapy; SA, surgery alone; TNM, Tumor Nodes Metastasis; LN：lymph node; Path:pathology ; MPV: mean palalet volume;

Table S3: cox regression Univariate and multivariate analysis for overall survival of patients with low mean palalet volume

| Low MPV OS |  | Univariate analysis |  |  | multivariate analysis |  |
| --- | --- | --- | --- | --- | --- | --- |
|  |  | HR(95%CI) | P |  | adj. HR_95CI | adj. P |
| age | ≤65 |  |  |  |  |  |
|  | ＞65 | 1.56 (1.24,1.98) | < 0.001 |  | 1.44 (1.12~1.85) | 0.005 |
| Gender | Male | 1 |  |  | 1 |  |
|  | Female | 0.73 (0.5,1.05) | 0.091 |  | 0.76 (0.52~1.12) | 0.162 |
| KPS | 90-100 | 1 |  |  | 1 |  |
|  | 70-80 | 1.16 (0.91,1.47) | 0.231 |  | 1.18 (0.91~1.51) | 0.209 |
| Number of LN | ≤15 | 1 |  |  | 1 |  |
| resected | ＞15 | 0.96 (0.74,1.23) | 0.727 |  | 0.82 (0.63~1.07) | 0.144 |
| Differentiation | Well | 1 |  |  | 1 |  |
|  | Moderate | 1.12 (0.79,1.6) | 0.522 |  | 1.12 (0.77~1.63) | 0.545 |
|  | Poor | 1.28 (0.9,1.81) | 0.168 |  | 1.29 (0.9~1.87) | 0.168 |
| Location | Upper | 1 |  |  | 1 |  |
|  | Middle | 0.97 (0.75,1.26) | 0.828 |  | 0.93 (0.71~1.22) | 0.603 |
|  | Lower | 1.16 (0.84,1.59) | 0.368 |  | 0.97 (0.69~1.35) | 0.854 |
| Lymphovascular | Yes | 1 |  |  | 1 |  |
| invasion | No | 0.71 (0.55,0.92) | 0.009 |  | 0.89 (0.67~1.17) | 0.391 |
| Neural invasion | Yes | 1 |  |  | 1 |  |
|  | No | 0.81 (0.62,1.06) | 0.118 |  | 0.95 (0.71~1.27) | 0.71 |
| Path T stage | T1 | 1 |  |  | 1 |  |
|  | T2 | 2.46 (1.11,5.47) | 0.027 |  | 2.14 (0.95~4.8) | 0.065 |
|  | T3 | 3.29 (1.55,6.99) | 0.002 |  | 2.85 (1.33~6.12) | 0.007 |
|  | T4a | 3.65 (1.65,8.07) | 0.001 |  | 2.93 (1.28~6.73) | 0.011 |
| Path N stage | N0 | 1 |  |  | 1 |  |
|  | N1 | 0.9 (0.46,1.77) | 0.762 |  | 0.86 (0.39~1.86) | 0.694 |
|  | N2 | 1.51 (0.77,2.99) | 0.233 |  | 1.38 (0.64~2.98) | 0.416 |
|  | N3 | 1.86 (0.91,3.76) | 0.087 |  | 1.57 (0.71~3.48) | 0.263 |
| Posttreatment | S alone | 1 |  |  | 1 |  |
|  | S+POCRT | 0.56 (0.43,0.73) | < 0.001 |  | 0.62 (0.47~0.83) | 0.001 |

Abbreviations:KPS, Karnofsky performance score; LVI, lymphovascular invasion; POCRT, postoperative chemoradiotherapy; SA, surgery alone; TNM, Tumor Nodes Metastasis; LN：lymph node; Path:pathology ; MPV: mean palalet volume;

Table S4: cox regression Univariate and multivariate analysis for overall survival of patients with high mean palalet volume

| high MPV |  | Univariate analysis |  |  | multivariate analysis |  |
| --- | --- | --- | --- | --- | --- | --- |
|  |  | HR(95%CI) | P |  | adj. HR_95CI | adj. P |
| age | ≤65 | 1 |  |  | 1 |  |
|  | ＞65 | 0.9 (0.69,1.17) | 0.422 |  | 0.93 (0.71~1.21) | 0.578 |
| Gender | Male | 1 |  |  | 1 |  |
|  | Female | 0.7 (0.5,0.98) | 0.037 |  | 0.7 (0.5~1) | 0.05 |
| KPS | 90-100 | 1 |  |  | 1 |  |
|  | 70-80 | 1.09 (0.82,1.44) | 0.549 |  | 1.14 (0.85~1.53) | 0.37 |
| Number of | ≤15 | 1 |  |  | 1 |  |
| LN resected | ＞15 | 0.76 (0.57,1.02) | 0.067 |  | 0.72 (0.54~0.98) | 0.035 |
| Differentiation | Well | 1 |  |  | 1 |  |
|  | Moderate | 1.12 (0.78,1.62) | 0.535 |  | 1.14 (0.78~1.66) | 0.502 |
|  | Poor | 1.16 (0.8,1.68) | 0.431 |  | 1.14 (0.78~1.67) | 0.507 |
| Location | Upper | 1 |  |  | 1 |  |
|  | Middle | 0.95 (0.72,1.26) | 0.742 |  | 0.95 (0.71~1.26) | 0.707 |
|  | Lower | 0.7 (0.47,1.05) | 0.085 |  | 0.65 (0.43~0.98) | 0.039 |
| Lymphovascular | Yes | 1 |  |  | 1 |  |
| invasion | No | 0.71 (0.52,0.96) | 0.028 |  | 0.97 (0.69~1.35) | 0.844 |
| Neural invasion | Yes | 1 |  |  | 1 |  |
|  | No | 1.03 (0.76,1.4) | 0.83 |  | 1.27 (0.92~1.76) | 0.139 |
| Path T stage | T1 | 1 |  |  | 1 |  |
|  | T2 | 1.77 (0.74,4.24) | 0.199 |  | 1.92 (0.79~4.62) | 0.148 |
|  | T3 | 2.66 (1.18,6.01) | 0.018 |  | 2.72 (1.19~6.22) | 0.017 |
|  | T4a | 3.04 (1.27,7.28) | 0.012 |  | 4.73 (1.88~11.91) | 0.001 |
| Path N stage | N0 | 1 |  |  | 1 |  |
|  | N1 | 1.16 (0.59,2.3) | 0.66 |  | 2.11 (0.95~4.72) | 0.068 |
|  | N2 | 2.25 (1.13,4.48) | 0.021 |  | 3.93 (1.75~8.83) | 0.001 |
|  | N3 | 2.57 (1.23,5.34) | 0.012 |  | 4.07 (1.68~9.83) | 0.002 |
| Posttreatment | S alone | 1 |  |  | 1 |  |
|  | S+POCRT | 0.9 (0.68,1.2) | 0.482 |  | 0.8 (0.6~1.08) | 0.153 |

Abbreviations:KPS, Karnofsky performance score; LVI, lymphovascular invasion; POCRT, postoperative chemoradiotherapy; SA, surgery alone; TNM, Tumor Nodes Metastasis; LN：lymph node; Path:pathology ; MPV: mean palalet volume;

Table S5: cox regression Univariate and multivariate analysis for overall survival of patients with low MPV in the validation group

| Low MPV |  | Univariate analysis |  | multivariate analysis |  |
| --- | --- | --- | --- | --- | --- |
|  |  | HR(95%CI) | P |  | adj. HR_95CI |
| age | ≤65 | 1 |  | 1 |  |
|  | ＞65 | 1.33 (0.56,3.13) | 0.518 | 1.25 (0.43~3.61) | 0.68 |
| Gender | Female | 1 |  | 1 |  |
|  | Male | 2.13 (0.95,4.78) | 0.067 | 3.63 (1.39~9.5) | 0.009 |
| KPS | 90-100 | 1 |  |  |  |
|  | 70-80 | 1.95 (1.04,3.65) | 0.038 | 1.8 (0.89~3.63) | 0.101 |
| Number of | ≤15 | 1 |  |  |  |
| LN resected | ＞15 | 1.95 (0.47,8.05) | 0.356 | 1.56 (0.34~7.27) | 0.568 |
| Differentiation | Well | 1 |  | 1 |  |
|  | Moderate | 0.58 (0.18,1.95) | 0.382 | 0.64 (0.18~2.31) | 0.491 |
|  | Poor | 1.12 (0.33,3.78) | 0.851 | 1 (0.26~3.9) | 0.997 |
| Location | Upper | 1 |  | 1 |  |
|  | Middle | 0.48 (0.16,1.47) | 0.2 | 0.66 (0.2~2.22) | 0.502 |
|  | Lower | 0.56 (0.2,1.59) | 0.275 | 0.96 (0.28~3.22) | 0.944 |
| Lymphovascular | No | 1 |  | 1 |  |
| invasion | Yes | 0.9943 (0.555,1.7813) | 0.985 | 0.89 (0.44~1.79) | 0.745 |
| Neural invasion | No | 1 |  | 1 |  |
|  | Yes | 1.64 (0.86,3.11) | 0.134 | 1.02 (0.47~2.19) | 0.959 |
| Path T stage | T1 | 1 |  | 1 |  |
|  | T2 | 1.01 (0.270,3.757) | 0.99 | 0.92 (0.23~3.77) | 0.911 |
|  | T3 | 1.7 (0.66,4.36) | 0.268 | 1.34 (0.44~4.07) | 0.6 |
|  | T4a | 1.76 (0.47,6.59) | 0.398 | 4.22 (0.69~25.74) | 0.118 |
| Path N stage | N0 | 1 |  | 1 |  |
|  | N1 | 0.85 (0.2,3.63) | 0.826 | 1.8 (0.23~13.91) | 0.574 |
|  | N2 | 1.47 (0.34,6.33) | 0.608 | 3.85 (0.44~33.35) | 0.221 |
|  | N3 | 2.74 (0.53,14.18) | 0.23 | 6.56 (0.63~68.07) | 0.115 |
| Posttreatment | S+POCRT |  |  |  |  |
|  | S alone | 2.36 (1.3,4.28) | 0.005 | 2.34 (1.19~4.61) | 0.014 |

Abbreviations:KPS, Karnofsky performance score; LVI, lymphovascular invasion; POCRT, postoperative chemoradiotherapy; SA, surgery alone; TNM, Tumor Nodes Metastasis; LN：lymph node; Path:pathology ; MPV: mean palalet volume;


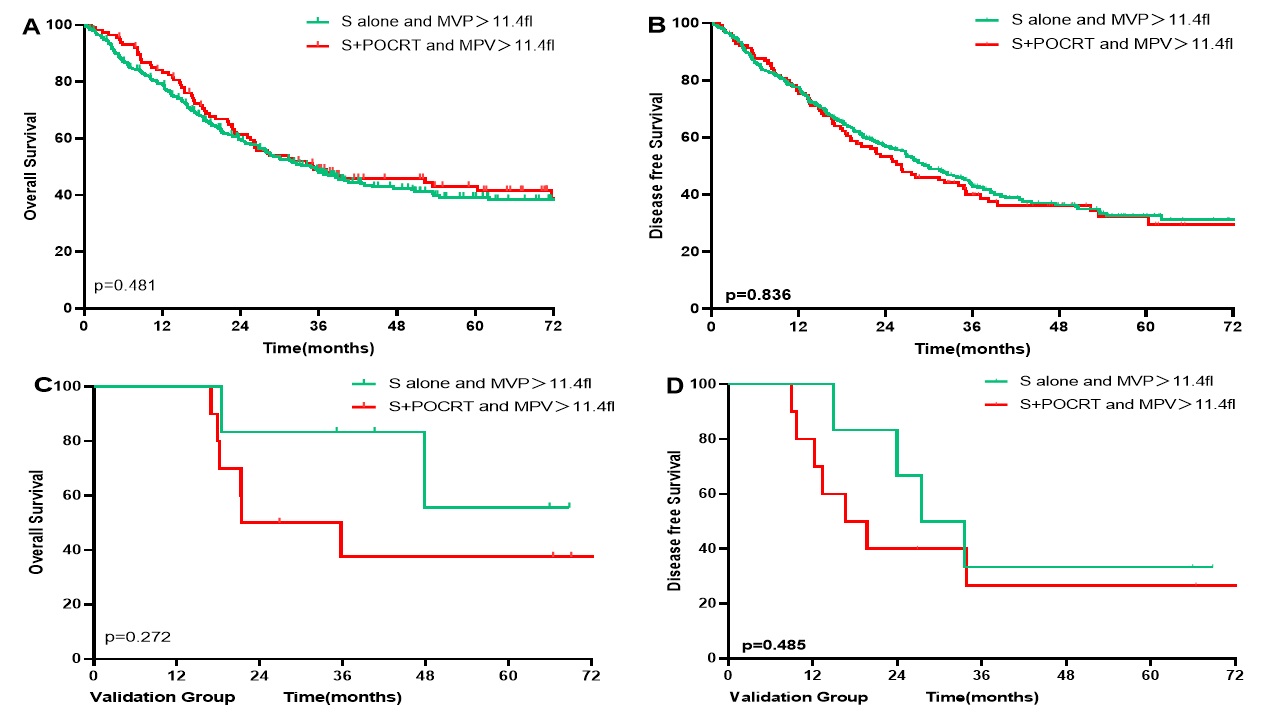


Figure S1 Comparison of overall survival and disease-free survival (A-B. The OS and DFS between POCRT and SA group for high MPV in develop population; C-D. The OS and DFS between POCRT and SA group for high MPV in the validation population).

Abbreviations: CI, confidence interval; HR, hazard ratio; POCRT, postoperative chemoradiotherapy; SA, surgery alone; MPV, mean platelet volume
